# Rapid in situ synthesis of polymer-metal nanocomposite films in several seconds using a CO_2_ laser

**DOI:** 10.1038/s41598-018-33006-9

**Published:** 2018-10-03

**Authors:** Kazuhiko Kashihara, Yuki Uto, Takashi Nakajima

**Affiliations:** Institute of Advanced Energy, Kyoto University, Uji, Kyoto 611-0011 Japan

## Abstract

We demonstrate the rapid *in situ* synthesis of polymer-metal nanocomposite films using a CO_2_ laser at 10.6 μm. The mechanism of our method is that the precursor of the metal nanoparticles, i.e., the metallic ions, is very rapidly reduced in the laser-heated polymer matrix without any reducing agent. Unlike other known laser-induced reduction methods using UV lasers, which produce radicals to promote reduction, the CO_2_ laser energy is mainly absorbed by the glass substrate, and the laser-heated substrate heats the polymer matrix through heat diffusion to promote reduction. The superiority of the use of CO_2_ lasers over nanosecond visible~UV lasers is also demonstrated in terms of the damage to the film. The developed method can be a new alternative to quickly synthesize a variety of polymer-metal nanocomposite films.

## Introduction

In recent years, the synthesis of various kinds of nanoparticles (NPs) and their applications have garnered great interest, as shown, for example, in ref.^1^. For the efficient and reliable use of NPs, a uniform dispersion of NPs is key. If NPs are used in a solution, then a surfactant can be conveniently introduced to ensure the uniform dispersion of NPs without aggregation. If NPs are dispersed in inorganic or organic matrices, such materials are called nanocomposites^2–5^, which have been of recent interest since the introduction of the various kinds of filler into the inorganic or organic matrices can result in the improvement of the mechanical, electrical, and optical properties of the matrix itself by the appropriate choice of the filler^6–9^.

There are many ways to synthesize organic (polymer) nanocomposites with metallic NPs as a filler^3,4^. The most straightforward way is to directly disperse metallic NPs into the polymer solution^10^. Another approach is to disperse NPs into the monomer solution and then induce polymerization^2^. An alternative method is to mix a monomer solution with a solution, which contains a precursor of the NPs, and then simultaneously induce reduction and polymerization to obtain NPs dispersed in a polymer solution^2,11^. After preparing a polymer solution with NPs, a nanocomposite film is easily obtained by spin-coating or casting methods. In addition to the above three processes, it is also possible to synthesize a polymer-metal nanocomposite film after making a polymer film, which contains a precursor of NPs, and then induce chemical^12,13^, photoinduced^14–18^, microwave^19^, or thermal reduction^20–23^ to produce NPs in the polymer matrix. These methods are called *in situ* reduction methods. Note that these *in situ* reduction methods require a relatively long time (typically tens of minutes to tens of hours) for the synthesis of nanocomposite films. Obviously, there is still room for us to seek a rapid and scalable method to synthesize *in situ* polymer-metal nanocomposite films.

**Figure 1:**
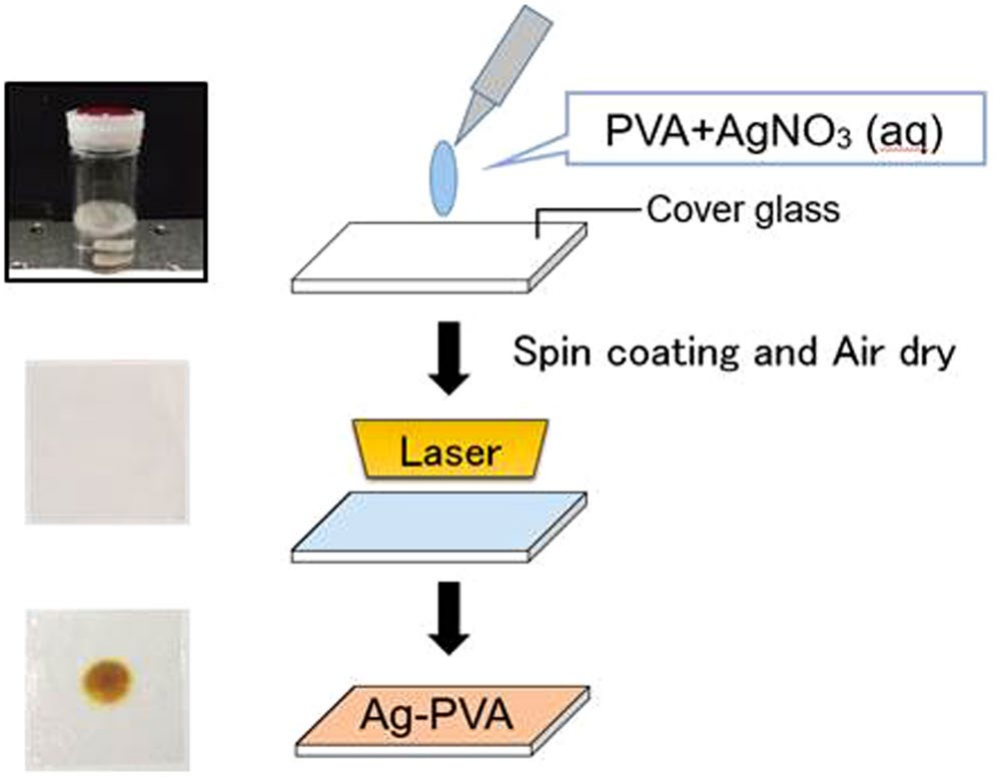
Illustration of the rapid *in-situ* synthesis of metal-polymer nanocomposite films using a CO_2_ laser.

In this paper, we demonstrate a rapid and scalable synthesis of polymer-metal nanocomposite films with a mid-infrared laser at a modest laser power. More specifically, we employ a CO_2_ laser at 10.6 μm and irradiate a spin-coated polymer (polyvinyl alcohol (PVA) or polyethylene glycol (PEG)) film containing the precursor of metal (Ag) NPs on a glass substrate, as shown in Fig. 1. Unlike the well-known photoinduced methods with a UV lamp or UV laser^14–18^ where photoexcited polymers or radicals of the additive are used to serve as a reducing agent, most of the CO_2_ laser energy is absorbed by the glass substrate rather than the polymer film itself, mainly because the glass substrate (thickness~0.15 mm) has a much larger absorbance than the spin-coated polymer film (thickness ~sub-μm) on it. Indeed, we measure the transmittance of the CO_2_ laser through the bare glass substrate and the free-standing polymer film to find that it is 0 and ~1, respectively. Thus, the polymer film on the laser-heated glass substrate effectively undergoes thermal annealing. In this paper, the synthesis of the Ag-PVA film is completed in only 10 sec, while the Ag-PEG film takes 10–40 sec, which may be compared with the other rapid synthesis methods developed under different contexts^24,25^. Moreover, we show that the synthesized Ag-PVA film can be made into a free-standing form^26–28^ by the removal of the film from the glass substrate. We would like to emphasize that, compared with the UV laser-based method^15^, the CO_2_ laser-based method we report in this work is an interesting alternative since it is suitable for the rapid and large-area synthesis of the nanocomposite films, whether they are on the glass substrates or in free-standing form.

## Experimental

### Materials

PVA (molecular weight (MW) ~60,000) and PEG (MW ~60,000 and ~500,000) are purchased from Sigma-Aldrich. Silver nitrate (AgNO_3_) and polystylene (PS) are purchased from Wako. All the chemicals are of reagent grade and used as purchased without any further purification.

### Lasers

For most of the experiments to be presented in this work we employ a CO_2_ laser at 10.6 μm (AL30P, Access Laser Co., peak power 60 W, pulse duration 100–400 μs depending on the laser power, repetition rate 2.5 kHz). Since the pulse duration is comparable to the pulse interval, which is 400 μs, it is nearly in the quasi-continuous-wave (CW) mode. To fabricate free-standing films we employ not only the CO_2_ laser but also the second and third harmonic of an Nd:YAG laser (INDI 30, Spectra Physics, maximum pulse energy 80 mJ at 532 nm and 70 mJ at 355 nm, pulse duration 8 ns, repetition rate 10 Hz) for comparison. The CO_2_ laser power is measured with a power metre (Pronto-250, Gentec-EO Co.), while the pulse energy of the Nd:YAG laser is measured with a thermal sensor (30A-P-SH-V1, Ophir) at the position of the polymer film. The laser beam diameter is ~8 mm (FWHM) with a Gaussian spatial profile at the position of the film for both lasers. As a result, the irradiated laser power on the film is different at different positions, which influences the film properties. Although it is possible to convert the Gaussian beam profile to a flat-top one using a beam shaper, we do not do this in this work. All the analyses at the film position are performed where the irradiated laser power is at its maximum.

### Synthesis of the Ag-PVA film

PVA (0.125 g) is mixed with 2 mL of highly purified water at room temperature under continuous stirring for 20 min. Then, the solution is heated to 95 °C for 45 min to completely dissolve the PVA. The PVA solution is mixed with a separately prepared solution, which contains 0.16 g of silver nitrate and 1 mL of water. Then, the mixed AgNO_3_-PVA solution is spin-coated on a microscope cover glass (22 × 22 × 0.15 mm) at 500 rpm for 5 sec followed by 4000 rpm for 10 sec. The AgNO_3_-PVA film is dried in air at room temperature for 30 min, and then it is irradiated with the CO_2_ laser or Nd:YAG laser at 355 or 532 nm under the chosen laser powers and durations.

### Fabrication of the free-standing Ag-PVA film

To fabricate a free-standing Ag-PVA film, a PS solution is prepared from 0.375 g of PS and 3 mL of toluene to spin-coat the cover glass at 1000 rpm for 10 sec as a sacrificial layer^4^. Then, the AgNO_3_-PVA solution is spin-coated on top of it. After the drying process, the AgNO_3_-PVA film with a sacrificial PS layer is irradiated with the CO_2_ laser at the chosen laser power and duration. After the CO_2_ laser irradiation, the synthesized Ag-PVA films with a PS layer are peeled off from the cover glass and dipped in a toluene solution for several seconds to dissolve the PS layer. Finally, using a wire ring of ~15 mm diameter, a free-standing Ag-PVA film is obtained.

### Synthesis of the Ag-PEG film

The process to fabricate an Ag-PEG film is almost the same as that of Ag-PVA described above. We employ PEG with two different MWs, MW~60,000 and 500,000. One gram of PEG is mixed with 2 mL and 4 mL of water, respectively. The respective PEG solutions are mixed with a separately prepared solution, which contains 0.16 g of silver nitrate and 1 mL of water. The AgNO_3_-PEG solutions are spin-coated on a cover glass, dried in air, and then irradiated with the CO_2_ laser.

### Characterization of the nanocomposite films

To characterize the fabricated polymer-metal nanocomposite films, we employ a compact CCD spectrometer (USB2000+, Ocean Optics), scanning electron microscope (SEM) (JSM-6500FE, JEOL) at 5 kV, and X-ray diffraction (XRD) (RINT-TTR III, Rigaku). For XRD, the diffraction angle is scanned with a speed of 5 °/min using a 10 kV micro-X-ray source obtained from the Co rotor target.

## Results and Discussions

### Film temperature

**Figure 2:**
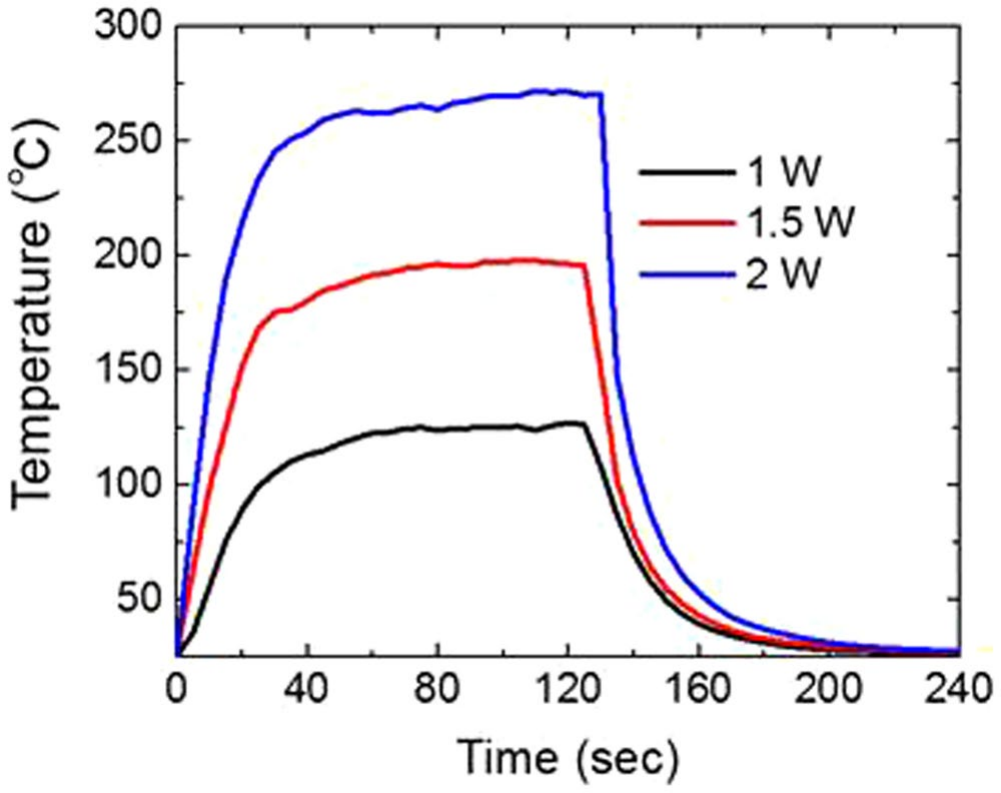
Temperature change in the PVA film on a cover glass substrate as a function of time. In this measurement, the laser is turned on and off at 0 and 120 sec, respectively.

To investigate the temperature change in the film during CO_2_ laser irradiation, we measure the film temperature. Since our CO_2_ laser is in the quasi-CW mode (i.e., pulse duration ~ pulse interval), we can conveniently use a thermocouple with a small head (~1 mm diameter) to measure the film temperature as a function of time. The results are presented in Fig. 2 at laser powers of 1, 1.5, and 2 W for an irradiation time of 120 sec. Upon laser irradiation, the film temperature rapidly increases and reaches a nearly steady-state temperature in 30 sec regardless of the incident laser power. Naturally, the steady-state temperature is higher for the higher laser power. When we turn off the laser, the film temperature rapidly cools down to the room temperature (~25 °C). Since the boiling and thermal decomposition temperatures of bulk PVA are approximately 230 and 300 °C, respectively, we can say that the appropriate range of CO_2_ laser power is 1–1.5 W.

### Ag-PVA films

**Figure 3:**
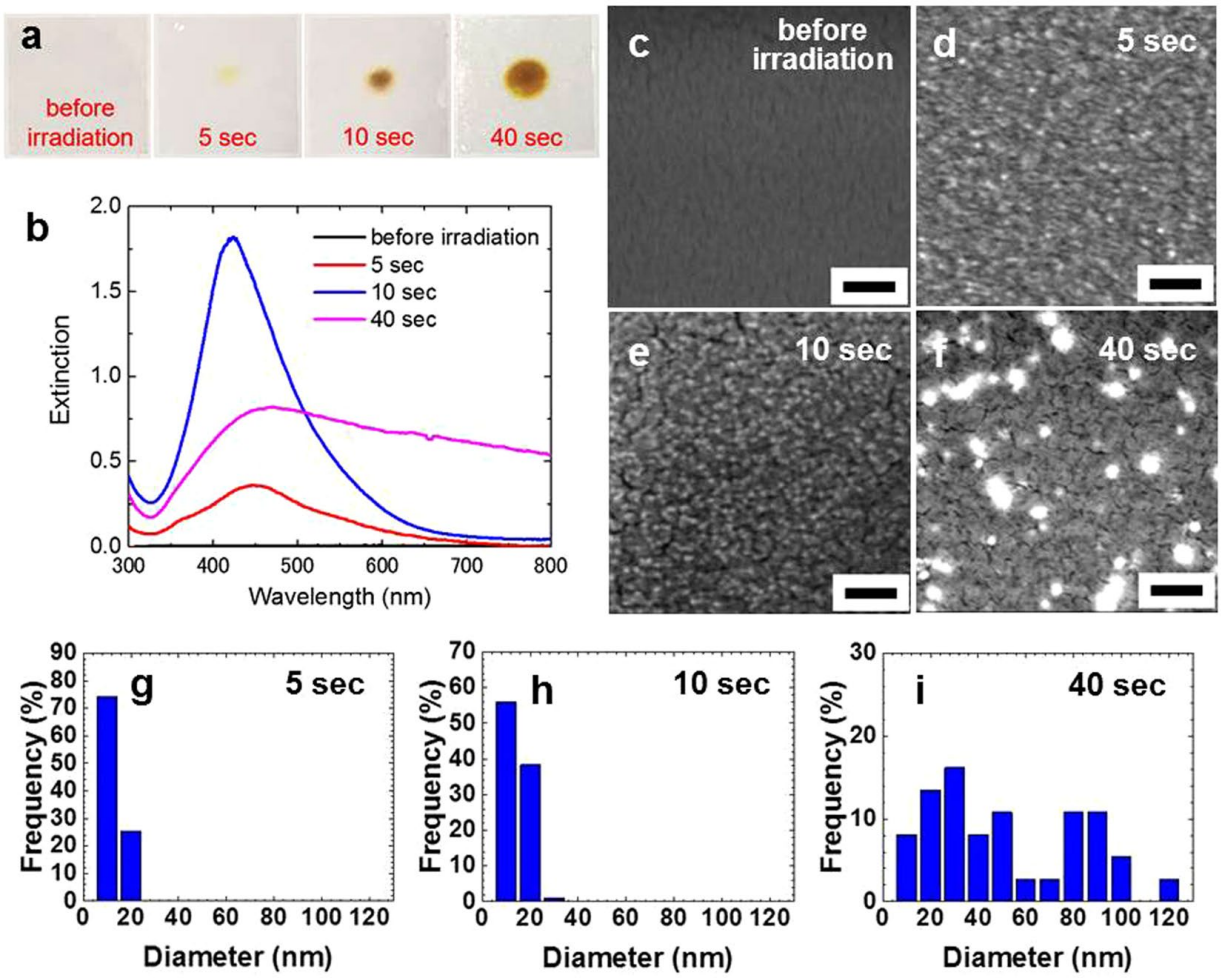
(**a**) Photos and (**b**) optical absorption spectra of the Ag-PVA films after the different irradiation times at the CO_2_ laser power of 1 W. (**c**) SEM image of the film before laser irradiation. (**d**–**f**) SEM images of the films and (**g**–**i**) the corresponding size distribution of Ag nanoparticles after CO_2_ laser irradiation at 1 W for 5, 10, and 40 sec, respectively. All scale bars represent 200 nm.

**Figure 4:**
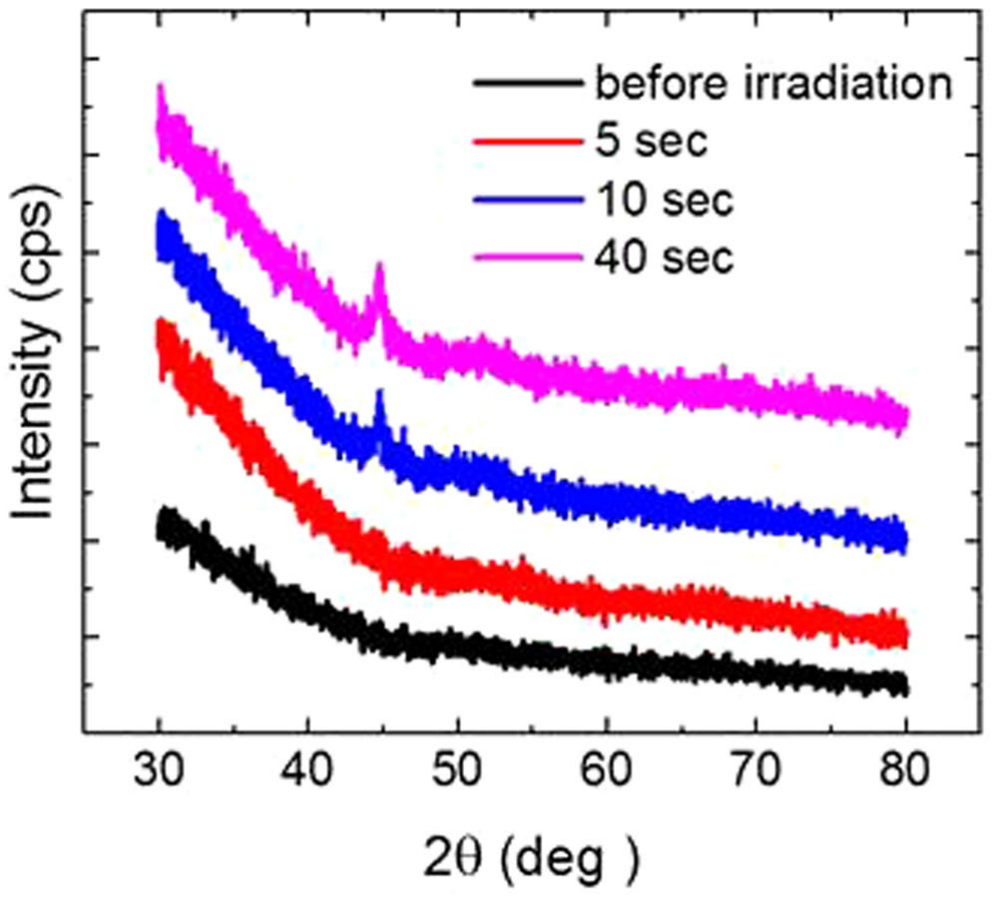
XRD spectra of the Ag-PVA films corresponding to Fig. 3.

**Figure 5:**
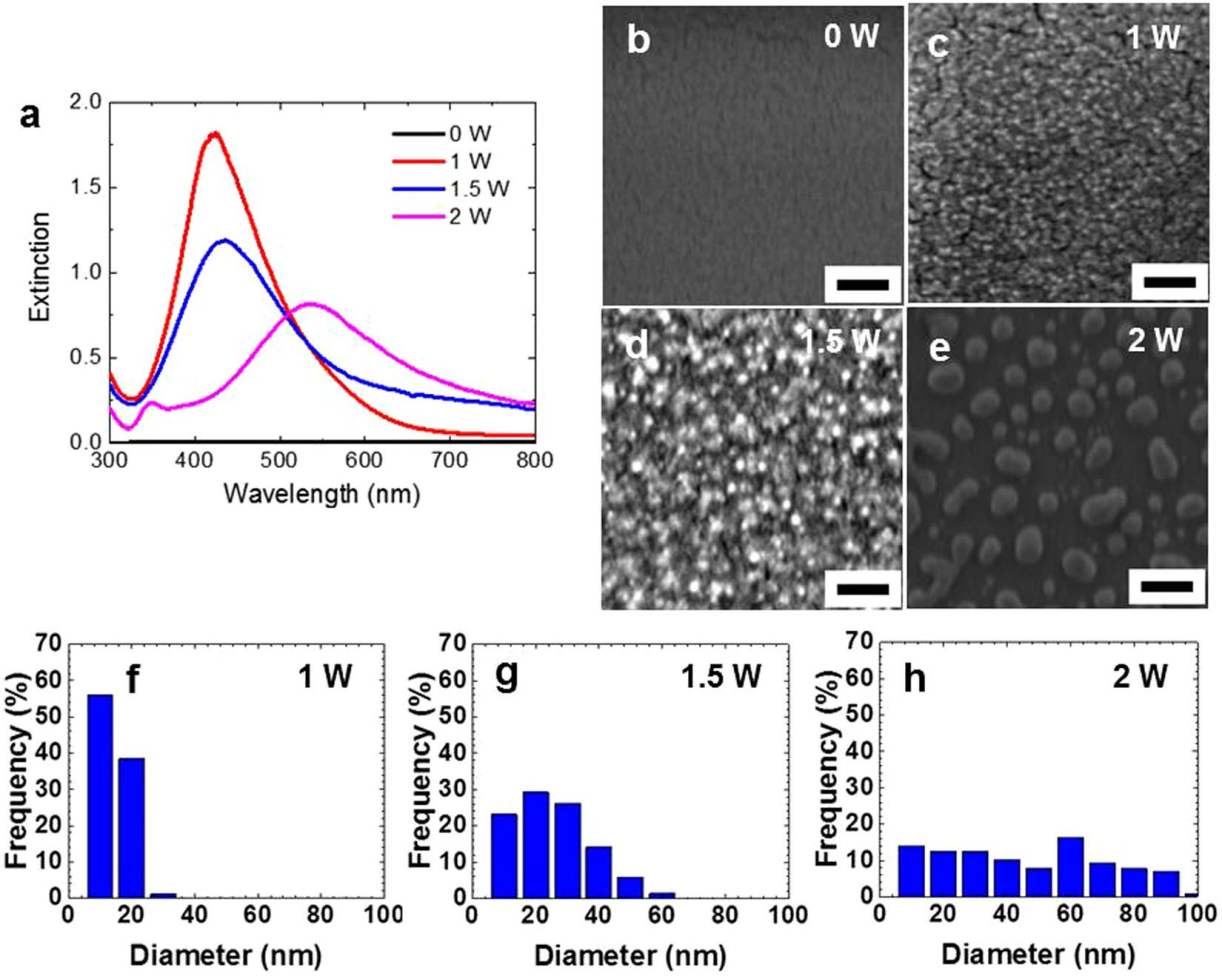
Characterization of the Ag-PVA films after 10 sec of irradiation at different CO_2_ laser powers. (**a**) Optical absorption spectra of the films. (**b**) SEM image of the film before laser irradiation. (**c**–**e**) SEM images of the films and (**f**–**h**) the corresponding size distribution of Ag nanoparticles after CO_2_ laser irradiation for 10 sec at 1, 1.5, and 2 W, respectivrely. All scale bars represent 200 nm.

We now irradiate the AgNO_3_-PVA film with a laser power of 1 W. Although the film is practically transparent before CO_2_ laser irradiation, the irradiated area of the film gradually turns yellow, as shown in Fig. 3a. The corresponding optical absorption spectra in Fig. 3b clearly indicate that the formation of Ag NPs in the PVA film takes place within a few seconds, and after irradiation for only 5 sec at 1 W, an SPR of the Ag NP is clearly observed. The SPR grows during the first several seconds of irradiation, and then from 10 to 20 sec, it stays nearly the same height and width (not shown here). After that, the height of the SPR gradually decreases and the tail extends to the longer wavelength side, and after the irradiation of 40 sec there is no clear tail on the long wavelength side. This change in the shape of the SPR implies the coalescence of NPs during the longer irradiation time, and this interpretation is confirmed by the SEM images (Fig. 3c–f) and the size distribution of the Ag NPs (Fig. 3g–i). The XRD spectra presented in Fig. 4 indicate additional evidence for the successful rapid *in situ* synthesis of Ag-PVA nanocomposite films. The crystalline size of the Ag NPs estimated from the peak width at 44° is 18.8 nm for the case of 40 sec irradiation at 1 W, which compares well with that of the 20 nm Ag-PVA film fabricated by the chemical reduction method^12^. (For the cases of 5 and 10 sec irradiation the peak heights in the XRD spectra are too small to reliably obtain the crystalline size.) Next, we fix the irradiation time to 10 sec and vary the laser power. The results are summarized in Fig. 5. By comparing the optical absorption spectra for laser powers of 1, 1.5, and 2 W (Fig. 5a), we can say that the 1 W laser power is sufficient for the 10 sec irradiation time in the sense that the use of higher laser power results in coalescence, as implied by the broader SPRs with long tails on the long wavelength side for laser powers of 1.5 and 2 W. This interpretation is again confirmed by looking into the SEM images (Fig. 5c–e) and the size distribution of the Ag NPs (Fig. 5f–h). During the 40 sec irradiation at 2 W the film temperature reaches 250 °C, and accordingly the property of the polymer matrix may change to some extent due to the partial thermal decomposition. This change is not a very serious problem, however, because we have chosen to employ a laser power of 1 W with the CO_2_ laser to synthesize the nanocomposite films.

### Free-standing Ag-PVA films

**Figure 6:**
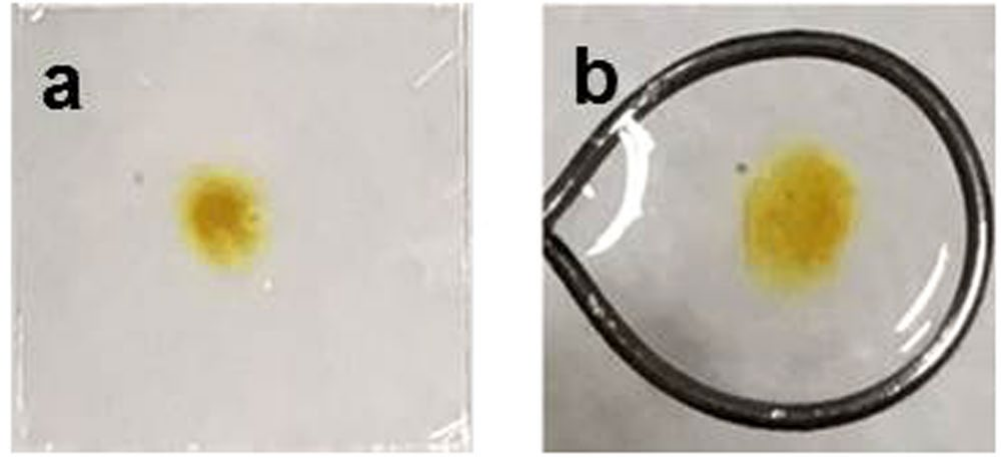
Photos of the (**a**) Ag-PVA film on a glass substrate and (**b**) free-standing Ag-PVA film, which are obtained after the 10 sec irradiation of CO_2_ laser at 1 W.

Now, we synthesize Ag-PVA films in a free-standing form with the procedure described above. After the CO_2_ laser irradiation onto the PS + AgNO_3_-PVA film for 10 sec at 1 W, we measure the optical absorption spectra to confirm the formation of Ag NPs in the PVA film, which can be alternatively and most conveniently confirmed by the change in the film’s colour, as shown in Fig. 6a. Then, we peel off the synthesized PS + Ag-PVA film from the glass substrate and dip it into a toluene solution^4^. The PS layer is dissolved into the toluene solution in a few seconds, leaving the Ag-PVA film alone. Finally, we capture the Ag-PVA film in the toluene solution with a wire ring of approximately 15 mm diameter to obtain the free-standing Ag-PVA film, as shown in Fig. 6b.

### Ag-PVA films by 355 and 532 nm lasers

**Figure 7:**
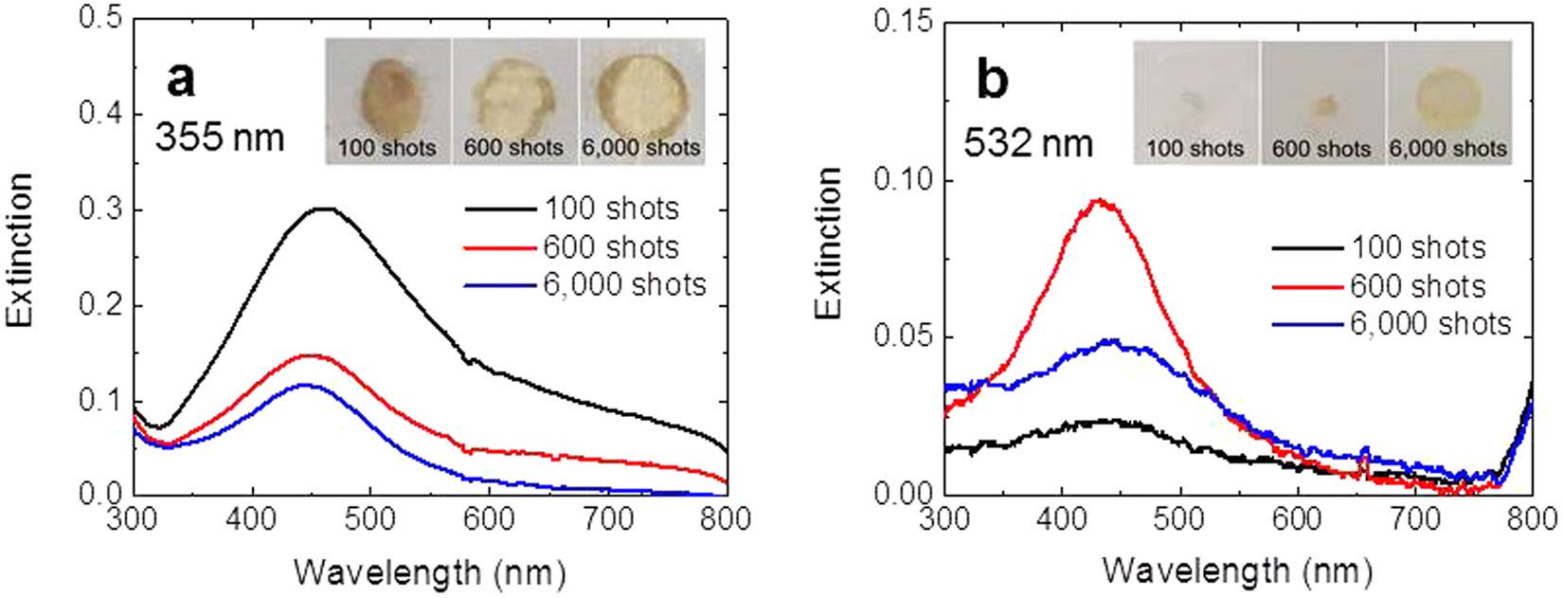
Optical absorption spectra of the synthesized Ag-PVA films with the (**a**) 355 nm and (**b**) 532 nm lasers for the different numbers of laser shots at 10 Hz. The employed laser fluence is 100 mJ/cm^2^ for both cases. The photos in the inset show the synthesized Ag-PVA films on glass substrates.

For comparison, we employ the third harmonic (355 nm) and second harmonic (532 nm) of an Nd:YAG laser to synthesize Ag-PVA films because it is one of the most commonly used lasers for the processing of various materials, and irradiate the AgNO_3_-PVA films at 10 Hz. Optical absorption spectra of the films after the irradiation of 100–6000 laser shots at the fluence of 100 mJ/cm^2^ are shown in Fig. 7. From the fact that the SPR appear by the irradiation of 355 and 532 nm lasers and it grows as the number of laser pulses increases, we can confirm that the Ag-PVA film is successfully synthesized with the 355 and 532 nm lasers even without any reducing agent^14,15^. However, the height of the SPR in the sample produced with the 355 nm laser is much smaller than that produced by the CO_2_ laser (Fig. 3b), and when the 532 nm laser is employed, the SPR is even smaller. This difference arises from the different photoabsorption mechanisms induced by the 355 and 532 nm lasers, which have a duration at 10 Hz of a few nanoseconds, and the CO_2_ laser with a duration of a few hundred microseconds at  2.5 KHz: The film irradiated by the quasi-CW CO_2_ laser stays at a high temperature for much longer time, which efficiently promotes the photothermal annealing needed to form Ag NPs. In contrast, the nanosecond 355 and 532 nm lasers can hardly induce the photothermal process and can only induce the photoexcitation of the polymers during the laser pulse, for which the irradiation of the 532 nm laser is less efficient than that of the 355 nm laser because the photon energy at 532 nm is too small to induce the relevant photoexcitation of the polymer molecules. A further increase in the laser fluence does not help since it damages the film. Actually, even at a fluence of 100 mJ/cm^2^, the film on the glass substrate is already damaged after a few hundred laser shots (inset photos of Fig. 7), and we cannot make a free-standing Ag-PVA film. We note that this kind of damage does not occur at all when we employ the CO_2_ laser at 1~2 W, probably because the pulse duration of the CO_2_ laser is very long and hence the heating is very mild. This finding demonstrates that the use of a CO_2_ laser is much more suitable than a pulsed Nd:YAG laser for fabricating metal-polymer nanocomposite films without damage.

### Ag-PEG films

**Figure 8:**
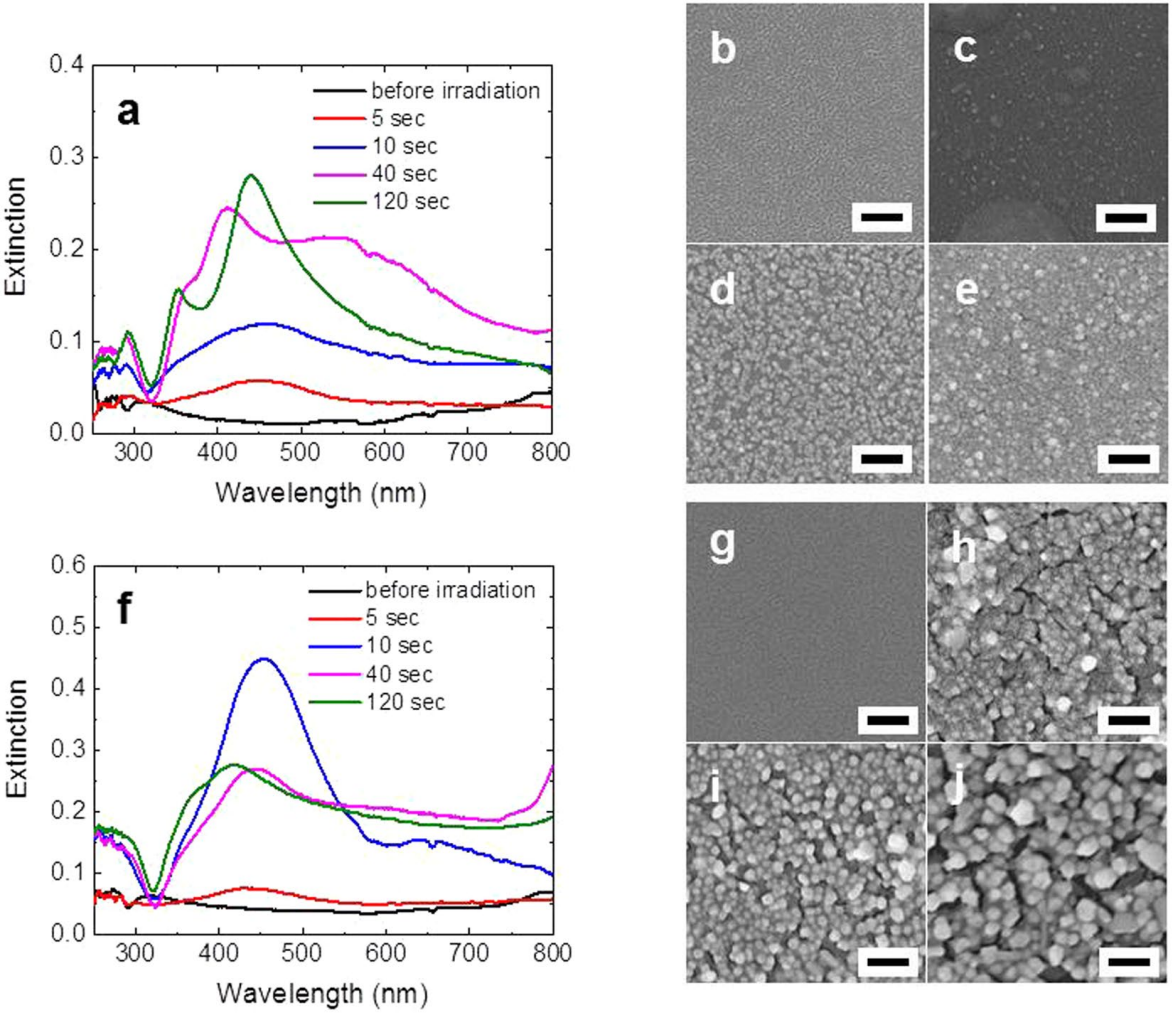
(**a**) Optical absorption spectra of the Ag-PEG (MW~60,000) films after different irradiation times at the CO_2_ laser power of 1W. (**b**) SEM image of the film before laser irradiation. (**c**–**e**) SEM images of the films after CO_2_ laser irradiation at 1 W for 10, 40, and 120 sec, respectively. (**f**–**j**) are similar to those of (**a**–**e**) but PEG with MW~500,000 is employed to fabricate the Ag-PEG films. All scale bars represent 200 nm.

Before closing this paper, we demonstrate that we can also synthesize Ag-PEG films using the technique we have developed in this work. Figure 8 shows the optical absorption spectra of the synthesized Ag-PEG films using PEG with MW~60,000 (Fig. 8a) and the SEM images before (Fig. 8b) and after laser irradiation (Fig. 8c–e). Similar results are presented in Fig. 8f–j for the Ag-PEG films using PEG with MW~500,000. Notably, the MW of PEG we employ in this work is much larger than that of PEG (MW~200 to 6,000) employed in the earlier works^29,30^ where the reduction of silver nitrate occurs in the PEG solution. For the Ag-PEG films using PEG with MW~60,000, SPR of Ag starts to appear due to the irradiation of CO_2_ laser at 1 W after only several seconds, and it grows as the irradiation time increases (Fig. 8a). Note, however, that it is still much smaller than that of the Ag-PVA film (Fig. 3b) after 120 sec of irradiation. After the longer irradiation times of 40 and 120 sec, a secondary peak is observed at 350 nm. This is a plasmon resonance of the bulk Ag. The spectrum after 40 sec of irradiation has a plateau region in the range of 400–560 nm, and this may suggest that the Ag NPs produced under this condition have strong polydispersity. For the optical absorption spectra of the Ag-PEG films using PEG with MW~500,000 (Fig. 8f), 10 sec of irradiation is sufficient to obtain the eminent SPR at 450 nm. For longer irradiation times, the tail in the range of >600 nm becomes almost flat, implying that many coalescence of Ag NPs occurs.

As the successful synthesis of Ag-PEG films with our method suggests, our method should be applicable to a variety of polymer films with a metal precursor. For instance, this technique could be used to synthesize Au-PVA^15^, Ag-PEG, Au-PEG^31^, Ag-PVP ((poly)vinylpyrolidone)^32,33^, Ag-PEDOT/PSS, and so on.

## Conclusions

We have demonstrated the rapid *in situ* synthesis of metal-polymer nanocomposite films in several seconds using a CO_2_ laser. The rapid formation of nanoparticles in the polymer matrix is confirmed by the optical absorption, SEM, and X-ray diffraction measurements. The role of the CO_2_ laser is to heat the glass substrate, which indirectly heats polymers in the film through thermal diffusion. Although the polymers (polyvinyl alcohol and polyethylene glycol) employed in this work have very small reducing power at room temperature, the heated polymers rapidly reduce Ag ions, and eventually Ag nanoparticles form in the polymer matrix over the course of several seconds. Therefore, our method is more similar to thermal annealing rather than UV laser annealing. The advantage of our method is that, without introducing any reducing agent, we can synthesize nanocomposite films in several seconds at a modest laser power density of ~1 W/cm^2^ with a compact commercial CO_2_ laser, and the process is scalable since a high-power CO_2_ laser for industrial purposes is readily available for the large-area synthesis of nanocomposite films. We have also shown that the use of a CO_2_ laser is very suitable for the synthesis of free-standing nanocomposite films. The new fabrication method of nanocomposite films developed in this work can be a new alternative for quickly synthesizing a variety of polymer-metal nanocomposite films.
